# Fluoroscopy-Guided Lumbar Puncture Training: Needs Assessment for Alternative Teaching Methods Including Simulation

**DOI:** 10.7759/cureus.113154

**Published:** 2026-07-22

**Authors:** Carina W Yang, Olga Pasternak-Wise, Donald R Cantrell, Saad Ali

**Affiliations:** 1 Radiology, University of Chicago Medicine, Chicago, USA; 2 Radiology/Neuroradiology, University of Chicago Medicine, Chicago, USA; 3 Radiology/Neurointerventional Radiology, Northwestern University Feinberg School of Medicine, Chicago, USA; 4 Radiology/Neuroradiology, Medical College of Wisconsin, Milwaukee, USA

**Keywords:** competency-based medical education, fluoroscopy-guided, image-guided procedures, lumbar puncture training, medical education satisfaction, neuroradiology procedure, procedural competency, procedural education, radiology simulation, resident survey

## Abstract

Introduction: A lumbar puncture (LP) is frequently performed to access the thecal sac for withdrawal of cerebrospinal fluid (CSF), most often for diagnostic analysis. Fluoroscopy-guided LP (FGLP) has gained popularity compared to traditional bedside LP, due to increasing patient obesity and associated lower rate of traumatic puncture due to fluoroscopic visualization of the needle. FGLP benefits patients with challenging spine anatomy or postoperative versus degenerative changes. To optimize the learning environment and decrease radiation exposure as well as inexperience-related errors, we conduct a needs assessment regarding current radiology resident FGLP didactics and consideration for alternative teaching methods.

Materials and methods: An anonymous survey was administered to radiology residents at the University of Chicago (UC) and Northwestern University (NU) regarding didactics received prior to the trainee’s first clinical FGLP. A total of 27 UC surveys were distributed, while there were 28 total at NU, with response rates of 85% and 71%, respectively. An initial pilot survey was conducted with three UC radiology residents who regularly assist with contrast simulation projects. Participant characteristics were as follows: UC: 34% female and 66% male, with an age range of 26 to 33 and ranging in postgraduate years 2 to 6; NU: 40% female and 60% male, with an age range of 25 to 35 and postgraduate training years 3 to 6. Participants were asked about the level of preparedness for the first and most recent FGLP attempt on a five-point Likert scale. The participants’ level of satisfaction with current training methods was assessed to determine the desire for alternative training.

Results: Five percent NU/0% UC survey participants were “extremely satisfied” with the current level of FGLP training, and 30% UC/20% NU students were “slightly satisfied.” The majority of trainees (91% UC/80% NU) expressed interest in additional training, including virtual simulation and mannequin training.

Conclusion: Through this initial survey, we demonstrate the need for an alternative method for teaching FGLP to radiology trainees, with eagerness to explore further training by virtual methods or mannequin simulation.

## Introduction

A lumbar puncture (LP) is a frequently performed, medically necessary procedure that accesses the thecal sac, allowing for withdrawal of cerebrospinal fluid (CSF) for several purposes, primarily a diagnostic procedure for fluid analysis or opening pressure measurement in the setting of infectious, inflammatory, or autoimmune processes, as well as to determine the presence or absence of subarachnoid hemorrhage or neoplastic cells. In addition, there are therapeutic purposes for LP such as withdrawal of larger volumes of CSF for treatment of idiopathic intracranial hypertension or in preparation for lumbar drain placement.

Compared with bedside LPs that are usually performed by non-radiologist clinicians, fluoroscopy-guided LP (FGLP) has gained popularity as the rate of obesity rises in the general population [1]. In fact, the prevalence of adult obesity and severe obesity is expected to continue to increase nationwide, with a high accuracy prediction that by 2030 nearly one in two adults will carry the diagnosis of obesity [2]. In addition, FGLPs have a lower rate of traumatic puncture compared to bedside LPs, due to visualization of the needle entering the spinal canal using direct fluoroscopy [3]. Although the impact of obesity on LP outcomes has not been extensively studied, data support a lower rate of traumatic puncture by FGLP compared to the bedside method [4,5].

FGLP is also advantageous in patients with challenging anatomy due to severe spondylosis, scoliosis, or prior spine surgery [6,7]. This procedure provides CSF access and contrast confirmation of thecal sac location for the administration of intrathecal chemotherapy. Diagnostically, FGLP can be utilized for intrathecal injection of iodinated contrast for fluoroscopic and computed tomography (CT) myelography or cisternography, especially when a patient is unable to undergo magnetic resonance imaging, or when functional evaluation of the thecal sac contents is clinically warranted. Consequently, LPs have increasingly been performed under image guidance with radiologists now being the dominant provider [8]. Rates of FGLP increased from 37.1% to 54.0% among sampled Medicare beneficiaries from 2004 to 2017, compared to a concomitant decrease in LPs being performed by most other physicians [9].

Traditionally, FGLP is demonstrated, mentored, and initially supervised on awake patients, which has served as the standard method of medical education for the procedure. This may not be equitable for all trainees who typically attempt to acquire FGLP competency during various neuroradiology rotations throughout the duration of the training program. FGLP procedure volumes vary based on patient needs, seasonal patterns of infectious disease, and hospital-dependent factors. Furthermore, the teaching supervision provided to radiology trainees to achieve this competency may range from a more senior trainee, a faculty member, or at times even an advanced practice provider specifically employed to perform basic neuroradiology procedures including FGLP.

To the authors’ knowledge, no prior literature is available assessing the current status of FGLP education or addressing potential deficiencies in achieving procedural competence. Our primary objective was to conduct an initial needs assessment study across the two largest radiology residency programs in Chicago, by way of a brief anonymous subjective and objective survey. Secondary objectives in the single survey opportunity for the trainees include evaluating the current status of radiology resident FGLP didactics, assessing residents’ satisfaction with existing available teaching methods specific to this particular neuroradiological procedure, and determining potential desire for alternative teaching methods, if any. The research hypothesis is that the surveyed radiology trainees in the two radiology residencies will deem the traditional teaching of FGLP inadequate, and that the needs assessment will demonstrate the residents’ expressed appetite for a greater variety of and updated training techniques ahead of performing their first clinical FGLP.

## Materials and methods

Following Institutional Review Board exemption, an anonymous, voluntary traditional hard copy survey (see the appendix) was administered to radiology residents at the University of Chicago Pritzker School of Medicine (UC; N = 23) and Northwestern University Feinberg School of Medicine (NU; N = 20) during a single regularly scheduled didactic conference. An initial pilot survey was conducted with three UC radiology residents who regularly assist with contrast simulation projects. A total of 27 UC surveys were distributed, while there were 28 total at NU, with response rates of 85% and 71%, respectively. Participant characteristics were as follows: UC: 34% female and 66% male, with an age range of 26 to 33 and ranging in postgraduate years 2 to 6; NU: 40% female and 60% male, with an age range of 25 to 35 and postgraduate training years 3 to 6. Surveys were returned to the residency coordinator at each institution, ensuring surveys would remain anonymous to the research team. The participants’ year of postgraduate training in radiology was recorded.

The survey inquired about previous FGLP training received prior to the trainee’s first clinical FGLP experience, if any, and queried the approximate total number of FGLP procedures performed to date, in particular independently. Participants were asked the level of preparedness for the first FGLP attempt on a 10-point Likert scale (“not prepared at all” to “completely prepared”), as well as their level of comfort in FGLP skills during their most recent FGLP. The participants’ level of satisfaction with current training methods was assessed, as was the need for potential alternative training, including specific named methods. Lastly, survey participants answered a pair of objective questions regarding basic FGLP procedural technique.

All categorical variables were compared using Fisher’s exact test due to small expected cell counts, utilizing the following tested assumptions: categorical variables, mutually exclusive groups, independent observations, and fixed marginal totals. Fisher's exact test requires no assumptions about expected cell counts and is appropriate for analysis of small sample sizes. All statistical analyses were conducted using IBM SPSS Statistics for Windows, Version 29.0 (IBM Corp., Armonk, NY, USA). As this is a novel study, with a lack of prior data, a priori analysis of statistical power cannot be accurately calculated.

## Results

**Figure 1:**
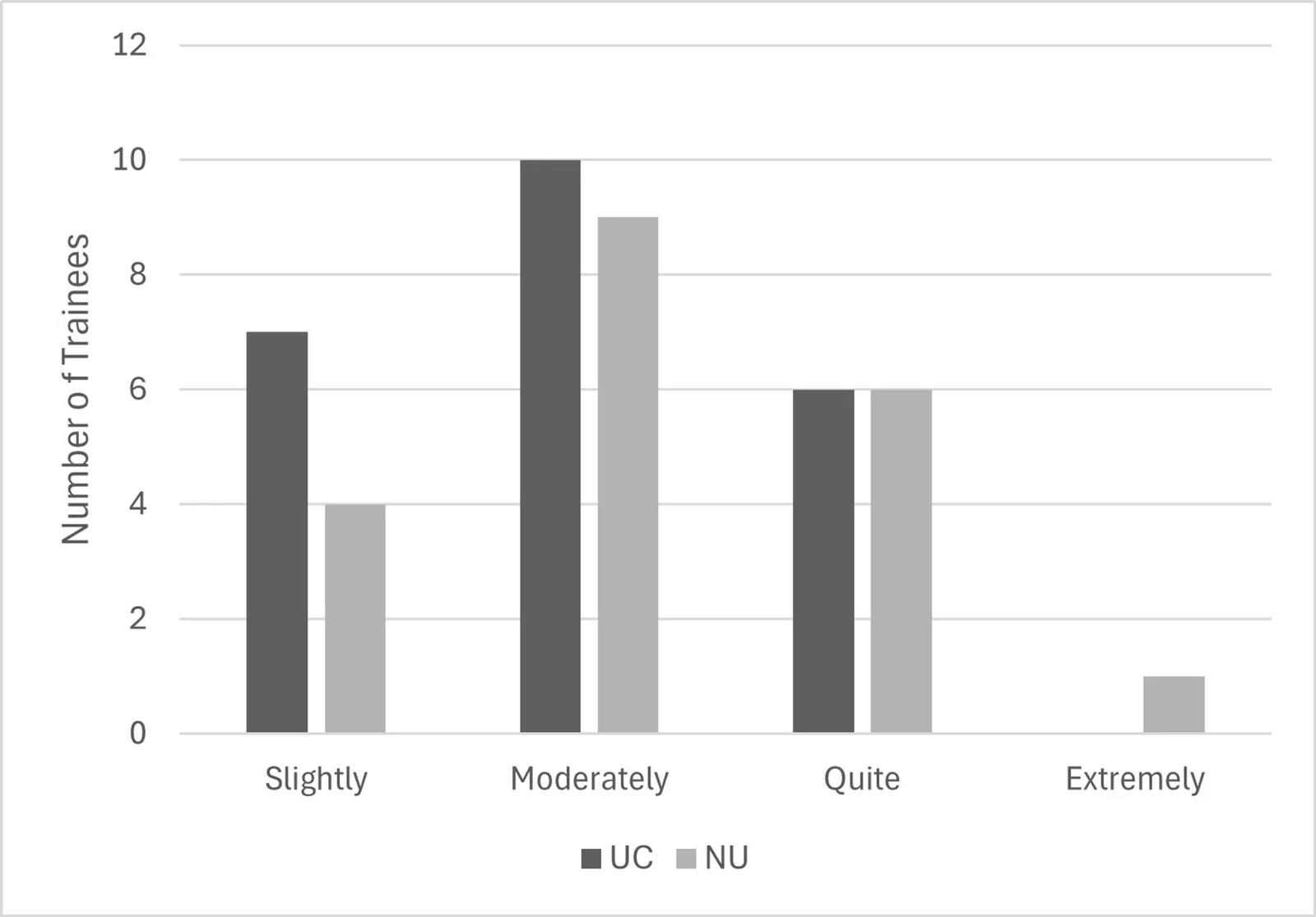
Level of satisfaction with current fluoroscopy-guided lumbar puncture (FGLP) training Of the surveyed residents at the University of Chicago (UC), none (0%) were extremely satisfied with the current teaching model, while of those surveyed at Northwestern University (NU), only one (5%) was extremely satisfied. The majority of the residents in each program expressed moderate satisfaction with their FGLP training thus far, with 7 (30%) UC and 4 (20%) NU residents feeling only slightly satisfied. Data have been represented as the number of radiology trainees (N). There is no significant difference in the degree of satisfaction with current FGLP training between the UC and NU trainees (p = 0.45, ordinal logistic regression).

Our results from the survey administered at two large peer institutions demonstrate that radiology residents express a relative lack of satisfaction with the current traditional methods of FGLP teaching, as seen in Figure 1.

**Figure 2:**
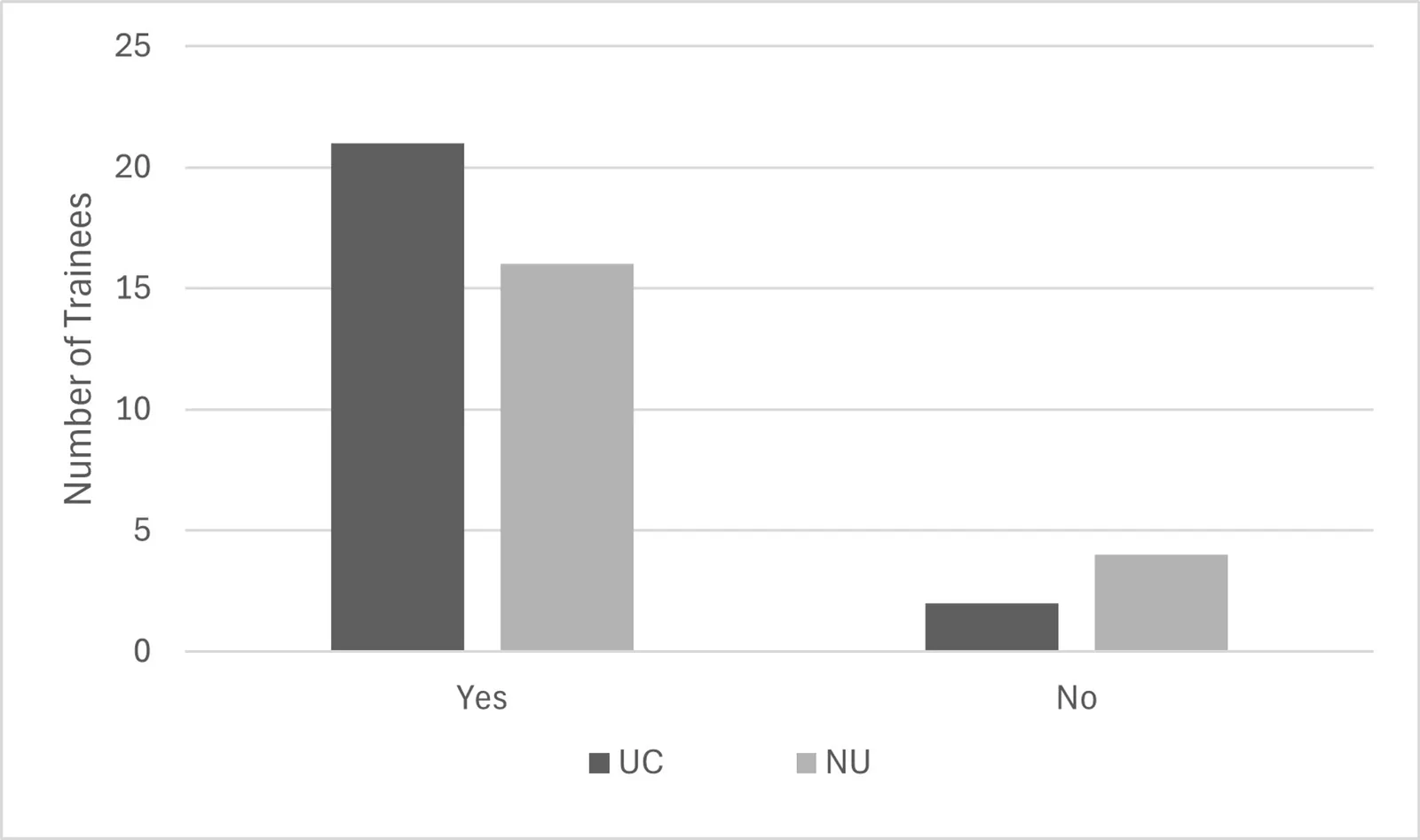
Need for additional FGLP training An overwhelming majority of radiology trainees at both UC and NU voiced a need for additional FGLP training (21 (91%) UC and 16 (80%) NU). Data have been represented as the number of radiology trainees (N). There is no significant difference in preferences for additional training methods between the two cohorts (p = 0.412, Fisher’s exact test). FGLP: fluoroscopy-guided lumbar puncture; NU: Northwestern University radiology trainees; UC: University of Chicago radiology trainees

**Figure 3:**
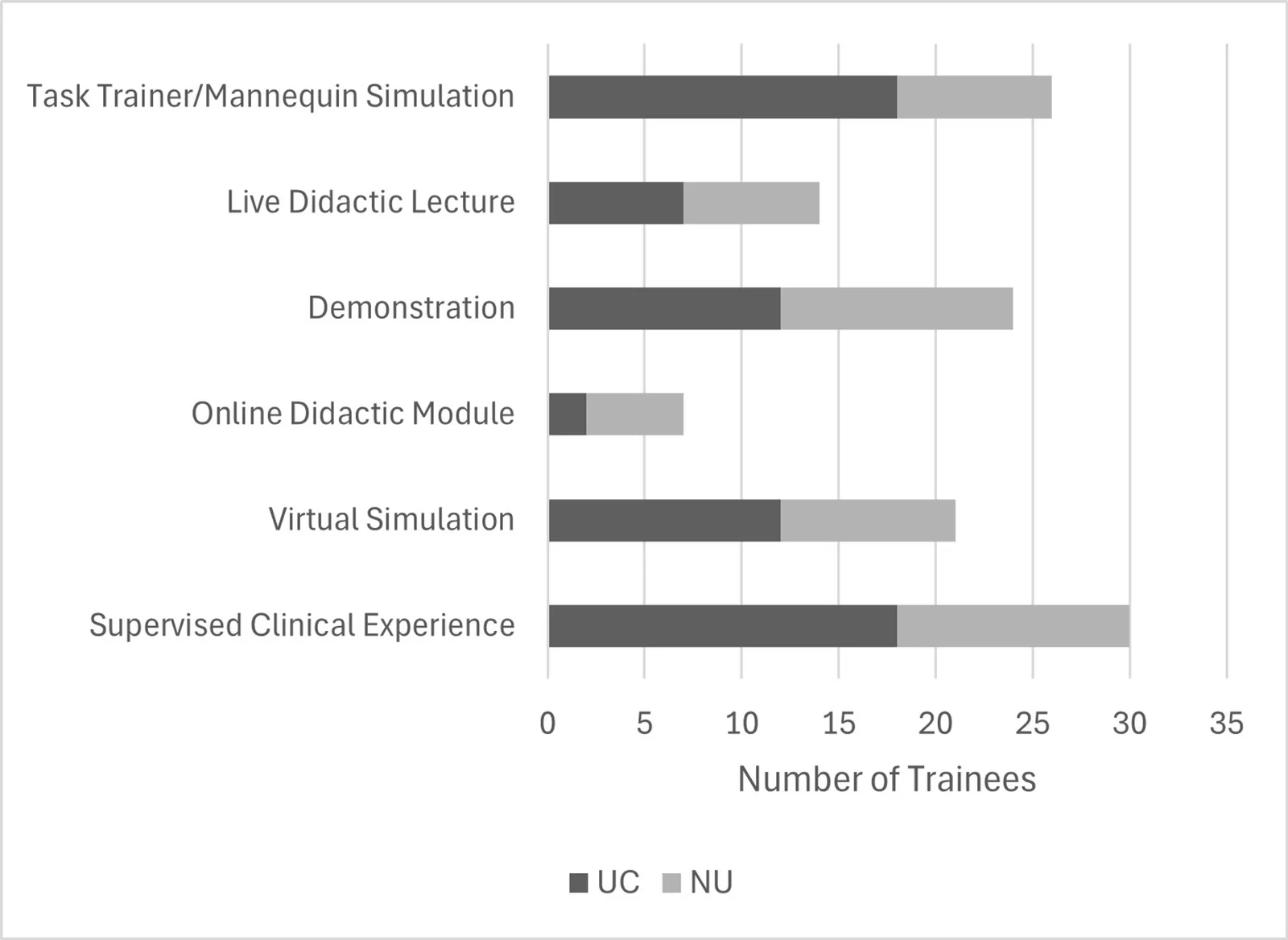
Interest in alternative teaching methods for FGLP training Trainees across the two largest Chicago academic radiology programs expressed a wide interest in training options for learning FGLP, with some newer and non-conventional methods. This includes 18 (78%) UC and 8 (40%) NU trainees seeking task trainer simulated teaching, as well as nearly 50% (12 (52%) UC and 9 (45%) NU) residents seeking virtually simulated techniques. Data have been represented as the number of radiology trainees (N). There is no significant difference in various alternative teaching methods for FGLP training between UC and NU trainees (p = 0.278, Fisher’s exact test). FGLP: fluoroscopy-guided lumbar puncture; NU: Northwestern University radiology trainees; UC: University of Chicago radiology trainees

Of the surveyed UC residents, none (0%) were “extremely satisfied” with the current teaching model, while only one (5%) NU resident was “extremely satisfied.” In addition, only seven (30%) UC and four (20%) NU residents were “slightly satisfied” with the current method(s) of teaching. There is no significant difference in the degree of satisfaction of current FGLP training between the UC and NU trainees (p = 0.45, ordinal logistic regression). However, an overwhelming majority of the residents sampled expressed interest in additional training, including simulation methods such as mannequin training (21 (91%) UC trainees and 16 (80%) NU trainees; Figures 2, 3). There is no significant difference in the various alternative teaching methods for FGLP training between UC and NU trainees (p = 0.278, Fisher’s exact test), nor in preferences for additional training methods between the two cohorts (p = 0.412, Fisher’s exact test). Based on our survey data and the enthusiasm expressed by the surveyed residents for additional procedural training, we see a need for alternate methods for teaching FGLP to radiology trainees.

## Discussion

FGLP competency

FGLP experience is typically gained only during neuroradiology rotations and is one of the more common image-guided procedures performed by radiologists. While the Accreditation Council for Graduate Medical Education does not denote a specific minimum requirement from neuroradiology rotations, resident experience in this subspecialty typically spans four to eight weeks per academic year in the first three years of radiology residency - when informally surveying other radiology residency program directors across Chicagoland and as suggested by the Association of Program Directors in Radiology “Rotation guides” [10]. The fourth and final year of residency generates greater variability in rotational experience, with many residents only receiving an additional four weeks of neuroradiology, often more focused on advanced imaging techniques rather than procedures.

FGLP competency involves understanding challenging anatomy including effects from degenerative and postoperative changes [11], and honing technical skill including efficient utilization of fluoroscopy. The traditional method of teaching FGLP relies on instructing trainees during real-time patient encounters, whereby the trainer discusses proper technique, models needle placement into the thecal sac using fluoroscopy, and ideally leads a discussion following the patient interaction. After procedural observation, the resident may attempt FGLP under direct supervision, with assistance and correction of the learner’s technique provided as needed. The learner must ultimately reproduce the series of tasks, albeit on varying patient anatomy, and familiarity with crucial tactile steps is only successfully gained as they acquire greater experience with subsequent patients.

This educational technique of “see one, do one, teach one” has been long used for procedural training in medicine; however, it may not serve as the most effective for producing confident FGLP operators. Multiple studies show that level of experience as well as type of patient body habitus has a significant effect on average associated fluoroscopy time [12-14], and greater competency therefore contributes to reduced radiation dose and other inexperience-related errors [8,15,16]. Learner anxiety is often high during initial attempts [16], and patients may perceive this anxiety as they are awake without sedation during the procedure. Lastly, there is concern regarding increased patient discomfort and minor complications if multiple attempts at FGLP are necessary, as can be common with new procedural learners [17].

Role of simulation

**Figure 4:**
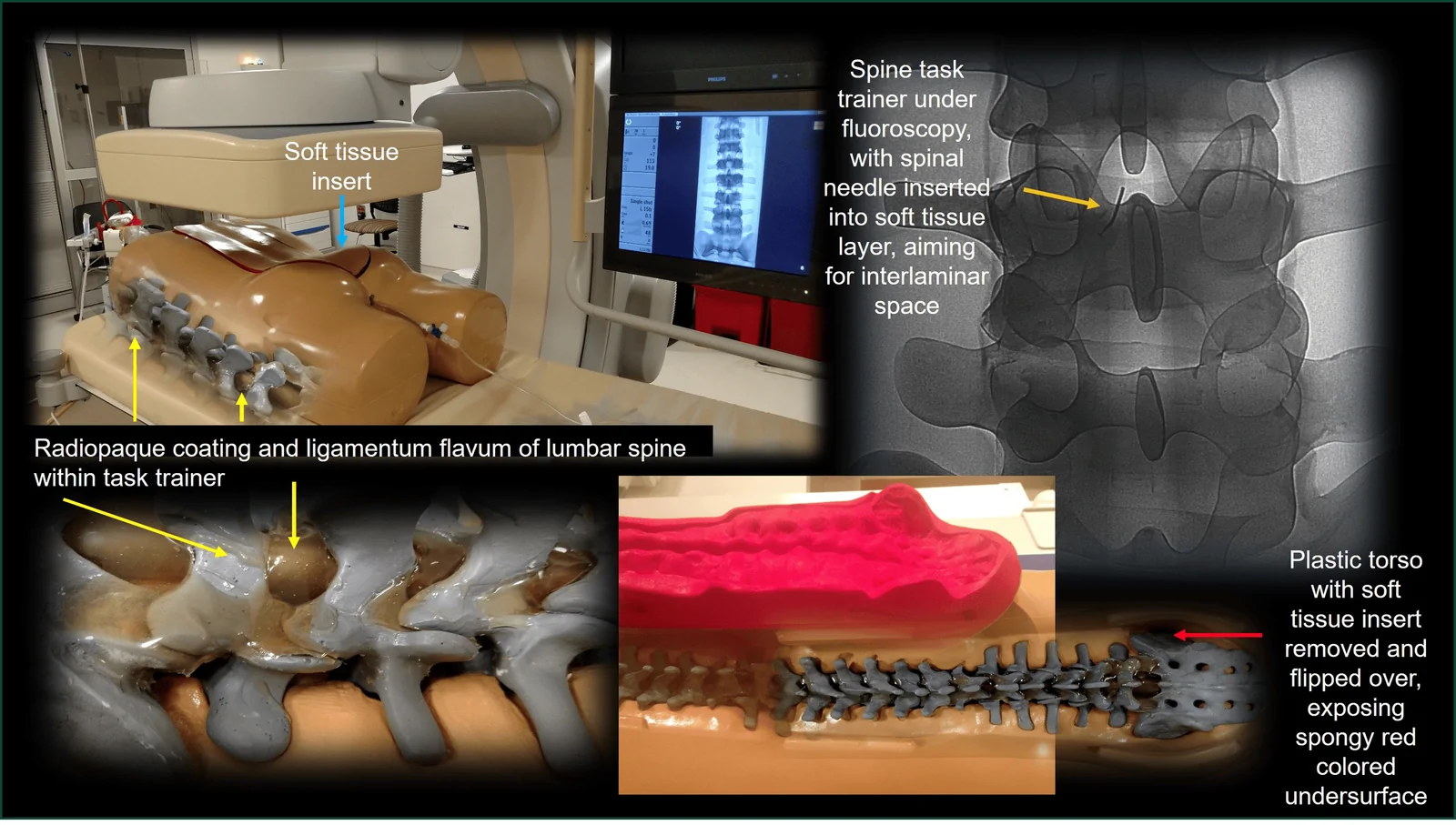
Custom lumbar task trainer for FGLP training This composite image demonstrates the custom-designed trainer with a Sawbones spine able to be visualized under fluoroscopy, as can be seen on the video screen of our dedicated spine procedural fluoroscopy suite. The gray spine model housed inside the coated foam simulated torso model can be seen as an inset at the bottom left of the photo. A silicone hollow tube filled with sterile water is threaded through the central spine canal of the model, allowing for a realistic attempt at FGLP on this mannequin. Figure independently created utilizing Microsoft PowerPoint (Microsoft Corp., Redmond, WA, USA). FGLP: fluoroscopy-guided lumbar puncture

Multiple studies confirm that retention of learned medical skills is greater if mannequin and/or simulation models are used, when compared to learners without exposure to such resources [16,18-20]. Simulation-based training has been widely adopted in other procedural specialties such as pediatrics and anesthesiology [16,19,21], and can likewise offer an opportunity for trainee improvement in FGLP skills with helpful feedback, prior to performance on patients. Although imaging phantoms are already embraced in some areas of radiology, they are not considered to be standard in current FGLP training during residency [12,22,23]. Developing new simulation-based training is associated with an initial cost, although this can be viewed as an investment in an important educational resource that can continue to be utilized in the future [16,19,20]. We previously designed and ultimately purchased a customized, barium-impregnated lumbar spine task trainer encased within a lower torso phantom, allowing for relatively high-fidelity, real-time procedural training utilizing direct fluoroscopy, as seen in Figure 4 [24].

**Figure 5:**
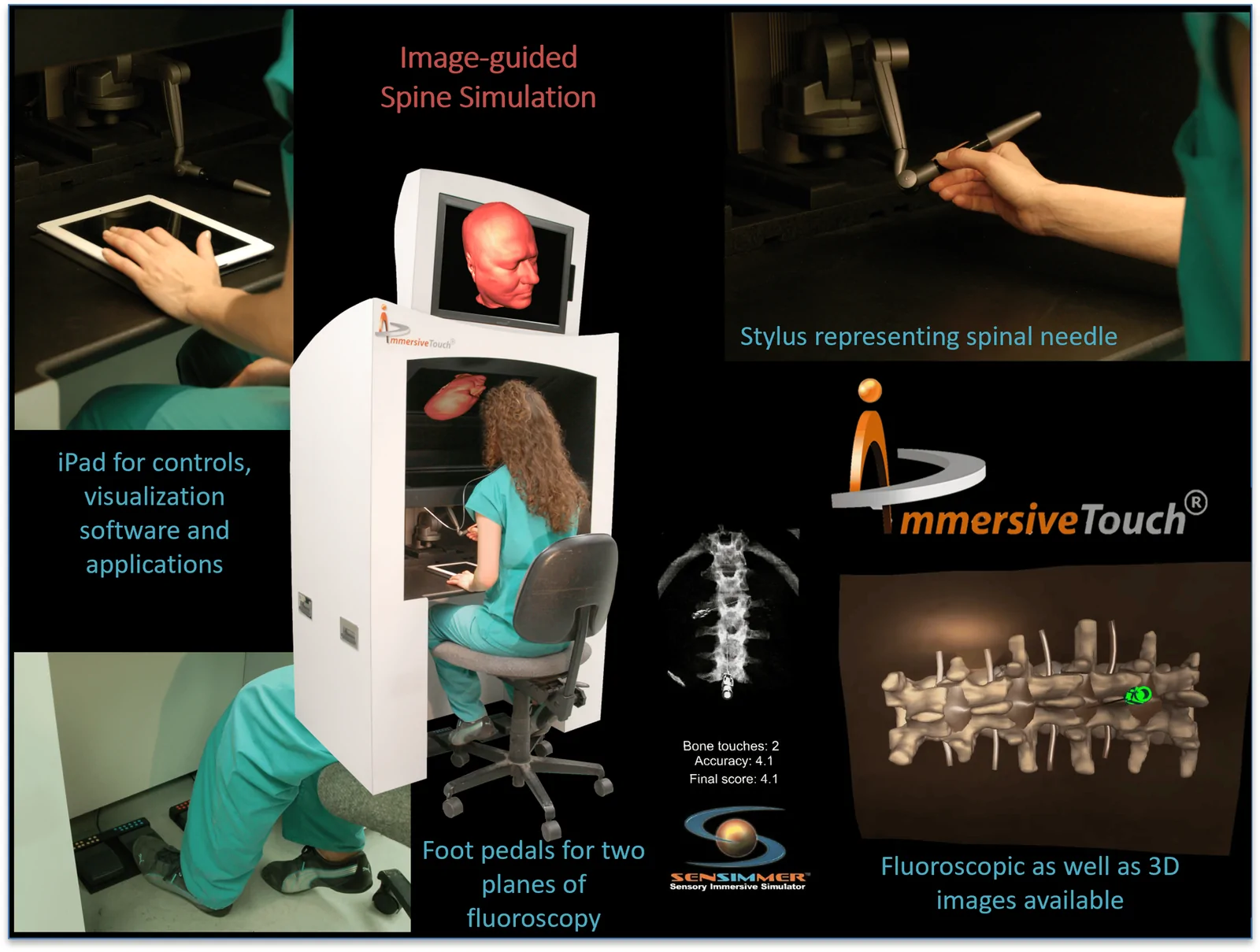
Virtual FGLP training module on the Immersive Touch system Screenshot of the software system utilized on the Immersive Touch platform (ImmersiveTouch Inc., Chicago, IL, USA), which serves to provide realistic haptic and visual cues and feedback to the learner as they attempt a virtual FGLP. By utilizing a handset control and mounted stylus, as well as goggles providing the ability to view the virtual instruments with respect to the relevant anatomy, the user can easily manipulate the virtual spine and needle for the facilitated understanding of complex three-dimensional spatial relationships. Figure independently created utilizing Microsoft PowerPoint (Microsoft Corp., Redmond, WA, USA). FGLP: fluoroscopy-guided lumbar puncture

Additionally, we piloted a virtual simulated program for FGLP, which provides direct haptic feedback to the user while performing the procedure on a three-dimensional visualized lower torso, all the while with the learner’s ability to manipulate the virtual spine by rotating the model or even cutting away at more superficial tissues potentially obscuring visibility of deeper structures, particularly the targeted thecal sac, as shown in Figure 5 [25].

The system can also provide helpful user statistical feedback regarding the number of needle passes, bone contact and distance from the target site, and “radiation dose” provided during the simulation process.

Procedural volumes and trends

FGLP procedural volumes, especially for trainees, can vary greatly based on institution. Our survey data demonstrate that UC residents were involved in as few as three and up to 60 FGLP, with the number of independently performed FGLP ranging from 0 up to 50 procedures. However, at NU, comparable survey data show results of 18 to 180 case FGLP involvement, with seven to nearly 100 independently performed. Some of this variability can be attributed to seasonal patterns of infectious disease influencing the ebb and flow of these procedures, as well as other differences in hospital-related characteristics such as overall neuroscience service-line or oncology volumes. For radiology trainees, their relative rate of invasive procedural tasks has steadily declined over the last few decades, as there is greater emphasis placed on efficient interpretation of continually increasing CT and magnetic resonance imaging volumes.

Utilizing national Medicare claims data collected from 1997 to 2016 as a relative measure of the changing distribution of radiological services performed by specialty trainees, Rosenkranz et al. noted that in 1997, various invasive procedures accounted for approximately one-third of radiology trainees’ relative work effort with FGLP included as one of the most commonly provided services; this decreased to approximately one-half of that in 2016, although FGLP remained the seventh most common invasive procedural service performed by radiology trainees [26]. Even more importantly, both diagnostic radiology and diagnostic radiology-interventional radiology residency curricula will require program vigilance to ensure they are receiving equitable training in image-guided procedures such as FGLP.

Increasing advanced practice provider support

Recently, advanced practice providers have increasingly supported the field of radiology, especially more recently, to aid in augmenting the national radiologist workforce shortage [27-29], including in the realm of procedures performed at busy academic institutions. With this influx of ancillary workforce, we must be increasingly vigilant in maintaining adequate learning opportunities for our radiology trainees, such that they will continue to adequately learn procedures and demonstrate competence before entering the field for independent practice. FGLP remains a common radiological procedure, even for those practicing more generally in private practice, as well as subspecialized academic neuroradiologists. With access to alternative didactics including simulation methods, radiology trainees will continue to benefit from repeated hands-on experience as desired.

Limitations

Our study harbors some limitations, including a relatively modest sample size with trainees from only two institutions, which may not be adequately representative of all radiology training programs in terms of their training experience. In addition, the study is limited by the sole utilization of assessment by subjective metrics rather than using more objective metrics, such as the number of failed FGLP or the total length of the procedure when performed by trainees who are less at ease with the procedure, although the latter would be challenging to measure given variations in the incidence of more senior residents, neuroradiology fellows, or even neuroradiology faculty ultimately assisting in the procedure as part of expected procedural workflow and for the safety and comfort of the patient. Additional inherent limitations of our data based on self-reported surveys potentially include recall and response biases (such as acquiescence bias, extreme responding, and demand characteristics) when participants reflected on their first FGLP experience. Again, data based on objective competency assessment would have been ideal.

## Conclusions

The results from our formal needs assessment suggest that there is a perceived lack of adequate trainee satisfaction with current medical education methods in achieving FGLP competency, which primarily involve “learning by doing.” The majority of surveyed radiology trainees clearly desire alternative ways of teaching this neuroradiological procedure and support the exploration of incorporating simulation-based training, such as virtual or mannequin simulation, into standardized FGLP teaching. These could potentially serve as key components of a more comprehensive FGLP curriculum ahead of the inevitable live-patient FGLP experience.

## Appendices

**Table 1:** Training for fluoroscopic-guided lumbar puncture (FGLP) needs assessment pre-survey PGY: postgraduate year

#	Question + answer options	
1	What year in radiology training are you?	
	A.	PGY-2
	B.	PGY-3
	C.	PGY-4
	D.	PGY-5
	E.	PGY-6
2	What kind of FGLP training have you had, if any? (free text)	
3	How many (please give your best estimate of a whole number) FGLP procedures have you:	
	A.	been involved in, in any manner? (free text) ______________________________________________________________
	B.	independently performed, successfully? (free text) ______________________________________________________
4	Think back to your first clinical FGLP. How prepared did you feel during the procedure on a scale of 1-10 (1 = not prepared at all, to 10 = completely prepared)? # ___	
5	At your most recent FGLP, how comfortable did you feel in your FGLP skills on a scale of 1-10 (1 = not comfortable at all, to 10 = extremely comfortable)? # ___	
6	How satisfied are you with current training methods?	
	A.	Not satisfied at all
	B.	Slightly satisfied
	C.	Moderately satisfied
	D.	Quite satisfied
	E.	Extremely satisfied
7	Would additional training in FGLP be helpful?	
	A.	Yes
	B.	No
8	What additional FGLP training would you be interested in? (please choose ALL that apply)	
	A.	Supervised clinical experience
	B.	Virtual simulation
	C.	Online didactic module
	D.	Demonstration ("See one, do one, teach one")
	E.	Live didactic lecture
	F.	Task trainer/mannequin simulation
9	Fluoroscopically, what anatomic space do you aim the spinal needle toward during FGLP?	
	A.	Intervertebral disk space
	B.	Foramen
	C.	Interlaminar space
	D.	Facet
10	What is the usual most cranial level of thecal sac access that should be safely attempted during FGLP?	
	A.	T12-L1
	B.	L1-L2
	C.	L2-L3
	D.	L3-L4
